# The Role of Transposable Elements in Sexual Development

**DOI:** 10.3389/fnbeh.2022.923732

**Published:** 2022-07-07

**Authors:** Vic Shao-Chih Chiang, Holly DeRosa, Jin Ho Park, Richard G. Hunter

**Affiliations:** College of Liberal Arts, Department of Psychology, Developmental and Brain Sciences Program, University of Massachusetts Boston, Boston, MA, United States

**Keywords:** epigenetic, retrotranspons, endogenous retroviral element, brain development, stress

## Abstract

Up to 50% of most mammalian genomes are made up of transposable elements (TEs) that have the potential to mobilize around the genome. Despite this prevalence, research on TEs is only beginning to gain traction within the field of neuroscience. While TEs have long been regarded as “junk” or parasitic DNA, it has become evident that they are adaptive DNA and RNA regulatory elements. In addition to their vital role in normal development, TEs can also interact with steroid receptors, which are key elements to sexual development. In this review, we provide an overview of the involvement of TEs in processes related to sexual development- from TE activity in the germline to TE accumulation in sex chromosomes. Moreover, we highlight sex differences in TE activity and their regulation of genes related to sexual development. Finally, we speculate on the epigenetic mechanisms that may govern TEs’ role in sexual development. In this context, we emphasize the need to further the understanding of sexual development through the lens of TEs including in a variety of organs at different developmental stages, their molecular networks, and evolution.

## Introduction

As part of the deep genome, transposable elements (TEs) make up roughly 50% of mammalian genomes and neuroscience researchers have only begun studying them intensively in the past decade (Bartlett and Hunter, 2018). They can mobilize around the genome and may be exapted to benefit their host through a variety of mechanisms (Hunter, 2020). To persist in a species, they insert themselves into the germline to facilitate sexual transmission (Dechaud et al., 2019). During development, TEs are crucial for totipotent zygote formation (Kruse et al., 2019). There are also several lines of evidence pointing to TE involvement in sexual development, including their biased presence in sex chromosomes, the sexual dimorphism of TEs, and their relevance to sexual development genes (Chatterjee, 2017). In this literature review, we first introduce TEs in terms of their classification. We then provide an overview of the different functions of TEs, followed by a review of their role in general development. We also reviewed disorders and diseases relevant to dysfunctional TEs, and the epigenetic regulation of TEs. Finally, we examined the role of TEs in contributing to biological sex differences.

## Transposable Elements

Many TEs are theorized to derive from ancient viral infections or small non-coding RNAs (ncRNAs), such as transfer RNAs. These theories have in part been supported by their taxonomic distribution integrated with the phylogenetics of shared cored proteins across species (Wells and Feschotte, 2020). While their discoverer Barbara McClintock referred to them as “controlling elements,” they have long been considered genomic parasites, junk DNA, and structural buffers between genes (Ohno, 1972; Orgel and Crick, 1980; McClintock, 1984; Hunter, 2020).

**FIGURE 1:**
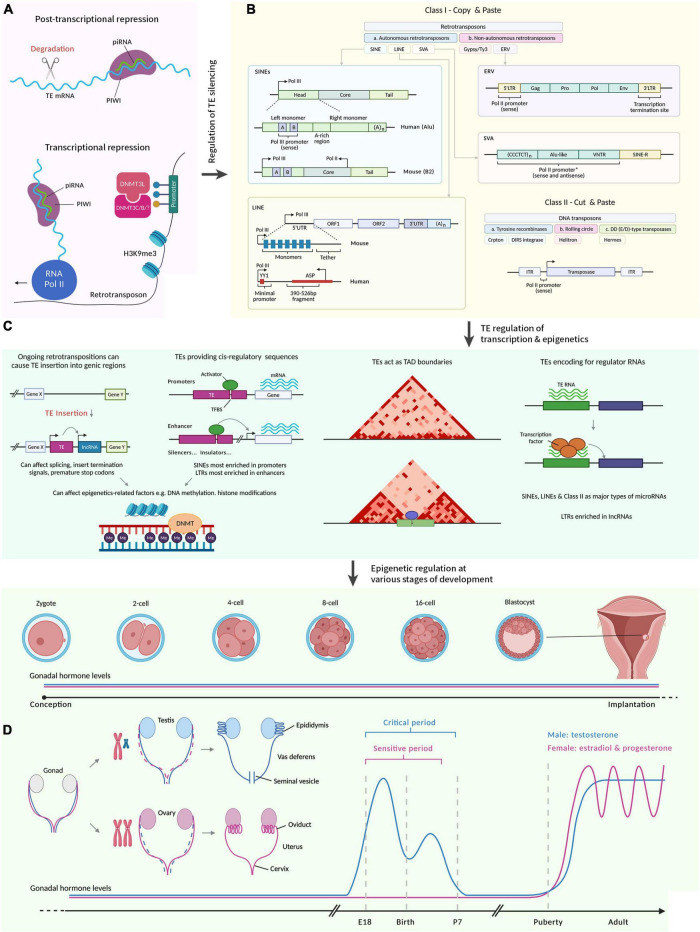
A summary illustrating how transposable elements regulate sexual development. Figures were adapted from various sources (McCarthy, 2020; Hermant and Torres-Padilla, 2021; Senft and Macfarlan, 2021; Almeida et al., 2022; Fueyo et al., 2022). Created with BioRender.com by Stephen Renjie Hu. **(A)** Regulation of TE silencing through post-transcriptional and transcriptional mechanisms. PIWI proteins are loaded with piRNAs to target sequence-specific TEs. The TEs are then degraded. Transcriptionally, piRNAs can recruit chromatin remodelers (e.g., DNMT) to silence TEs. **(B)** There are broadly two classes of TEs. Class I refers to retrotransposons that reverse transcribe before integration (“copy and paste”). Class II refers to DNA transposons that are excised and inserted (“cut and paste”). Class I is further divided into LTRs and is mostly ERV in mammals. ERV contain two LTRs that flank ORFs, which encode viral proteins. Class I TEs that lack LTRs are LINEs, SINEs (require LINE proteins to mobilize) and SVA (primate-specific). Boxes represent the different parts of the elements. L1 contain two ORFs, which encode retrotransposition proteins and are flanked by UTRs. There is also an adenine tail of variable length in the 3′ end. Alu consists of two monomers flanking an adenine-rich region and an adenine tail of variable length in the 3′ end. A and B boxes are a bipartite promoter for Pol. SVA contains a variable number of hexamer repeat in the 5′ region, followed by an Alu-like region with a variable number of VNTR, and followed by SINE-R. Class II encode a transposase required for their transposition and is flanked by two ITRs. **(C)** TEs regulate transcription and epigenetics. Ongoing retrotranspositions can cause TE insertion into genic regions. This insertion can be regulatory to transcribe previously transcriptionally inert sequences. It can also affect the splicing of existing genes transcribed, insert termination signals, and premature stop codons. TEs can provide *cis*-regulatory sequences including enhancers, promoters, silencers, and insulators. In promoters, TEs provide TFBS to influence transcription. SINEs are most enriched in promoters, and LTRs are most enriched in enhancers. Both TE insertion and presence in *cis*-regulatory sequences can affect epigenetic-related factors including DNA methylation and histone modifications. The 3D chromatin structure can be regulated by TEs, for example, by providing a TFBS for chromatin remodelers, which could lead to new boundaries between TADs. The dark-red triangles are TAD from Hi-C maps. TEs encode regulatory RNAs that for example, can interact with transcription factors to modulate gene expression. These regulatory RNAs include lncRNA and microRNAs. SINEs, LINEs, and Class II make up major types of microRNAs, whereas LTRs are enriched in lncRNAs. **(D)** TE regulation of transcription and epigenetics can affect various stages of development. Due to the diversity of sexual development across species, we focus here on humans and rodents. Embryogenesis occurs from a one-celled zygote that develops during the pre-implantation germinal stage to cleave into a multicellular embryo. Blastocyst forms and implants in the uterus. We then skip to sexual differentiation. Both the Müllerian (pink) and Wolffian (blue) ducts are present in the embryo. XX embryo retains the Müllerian ducts which differentiate into female reproductive tracts (oviduct, uterus, cervix etc.). XY embryo retains the Wolffian ducts which differentiate into male reproductive tracts (epididymis, vas deferens, seminal vesicles etc.) due to the SRY gene on the Y chromosome. In male rodents, there is a critical period (during late gestation to early postnatal stages) that coincide with exposure to testosterone (blue graph), which ends when removing testosterone does not affect masculinization. Complementary to this, female rodents have a sensitive period (still inconclusive when this starts and ends) which ends when exogenous testosterone does not disrupt feminization. During puberty, gonadal hormone levels rise again for both males and females. piRNA, piwi-interacting RNA; PIWI, P-element Induced WImpy testis; TE, transposable elements; DNMT, DNA methyltransferase; mC, cytosine methylation; Pol, polymerase; H3K9me3, histone 3 lysine 9 trimethylations; SINE, short interspersed elements; LINE, long interspersed elements; SVA, variable-number tandem-repeat Alu; ERV, endogenous retroviruses; L1, long interspersed elements 1; UTR, untranslated region; ORF, open reading frame; YY1, Yin-Yang 1; ASP, antisense promoter; Gag, group-specific antigens; Pro, protease; Env, envelope protein; LTR, long terminal repeats; C, cytosine; T, thymine; VNTR, variable number of tandem repeats; R, sequence of retroviral origin; ITR, inverted terminal repeats; A, A box or adenine; lncRNA, long non-coding RNA; TFBS, transcription factor binding sites; TAD, Topologically associating domain; Me, methylation; SRY, sex-determining region Y.

The two classes of TEs are retrotransposons and DNA transposons which can be characterized based on their mechanism of movement (Lapp and Hunter, 2016; Figure 1B). DNA transposons move through mechanisms that are non-replicative, or cut and paste (Ahmadi et al., 2020). DNA transposons are more common across species (Ali et al., 2021), but it is unclear if this is due to their smaller size. They encode a transposase that catalyzes their move into a new target site (Grassi et al., 2019). Retrotransposons insert their sequence into new genomic areas in a copy and paste fashion, through reverse transcriptase and transposases encoded by active retrotransposons. There are three classes of retrotransposons, namely, Long Interspersed Nuclear Elements (LINEs), Short Interspersed Nuclear Elements (SINEs), and Endogenous Retroviruses/Long Terminal Repeat elements (ERV/LTR). They are classified either as autonomous, which are self-sufficient to move, or the alternative, which is non-autonomous and requires the presence of active autonomous retrotransposons in the host genome (Ahmadi et al., 2020). LINEs are 6–8 kb in length and encode reverse transcriptase and endonuclease (Jurka et al., 2007; Lapp and Hunter, 2019). In contrast, SINEs are much shorter and do not encode the transposition machinery, and therefore rely on the autonomous LINEs for their machinery. ERV/LTR are similarly non-autonomous to SINEs and have a much more recent evolutionary origin than the other two types of retrotransposons. The specific transposition mechanisms of TEs have been reviewed (Wells and Feschotte, 2020), but still require a great amount of exploration.

## Molecular Functions of Transposable Elements

It has long been known that TEs can be harmful through the production of deleterious mutations and genome instability. However, exaptation may have occurred to confer benefits to the organism (Hunter et al., 2013, 2015; Grassi et al., 2019; Hunter, 2020). This genetic variation differs from other mutagenic mechanisms. TE insertions are often functional, as they can create promoter elements, regulatory sequences, splicing regulators and even protein-coding sequences (Payer and Burns, 2019; Figure 1C). Furthermore, TE insertions can promote new transcription factor (TF) binding sites, as well as coopt new enhancer elements and nuclear receptor response elements. These include binding sites for architectural proteins which confer widespread contribution to the dimensionality of the genome, as have been supported in computational models and validated through CRISPR-Cas9 experiments (Choudhary et al., 2022).

In addition to acting as *cis*-regulatory elements, TEs can rearrange 3′-untranslated regions of mRNA, shuffle exons between genes, move the entire gene to another site, and even render pseudogenes functional (Hunter, 2020). In fact, research has shown that TEs encode 20% of binding sites for TFs and generated 18–31% of transcription start sites (Pehrsson et al., 2019). In human and mouse genomes, a particular family of TEs are over-represented in the binding sites of certain transcription factors, demonstrating this relationship to be specific (Bourque et al., 2008). While a systematic database that maps specific TEs to affect the binding sites for specific TFs is currently lacking, several clues can be derived from their evolutionary origins to infer this relationship and could help shape future endeavors to construct this database. For instance, if the TE-derived TF binding sites arose for TFs present in both germ cells and somatic cells, then these TEs would likely affect binding sites for ubiquitous TFs like sex-determining region Y (SRY)-box TF 2, Nanog Homeobox, and estrogen-related receptor beta (Fueyo et al., 2022). In the case that the TF binding sites from TEs emerged from retroviral hijacking in immune cells, these would likely target those TFs engaging in innate immunity (Fueyo et al., 2022). TF binding sites from TEs may likewise come from mutations after genomic insertion, which may target ultra-conserved TFs primarily in somatic cells. This could be because of the length of evolutionary timeline required, if we assume ancient TEs prioritized germ cells for vertical transmission (Fueyo et al., 2022).

Aside from TF binding sites, TE sequences are present in a substantial fraction of microRNAs (miRNAs) and long non-coding RNAs (lncRNAs), which are known to be critical for physiological functions (Ali et al., 2021). For a comprehensive overview of how TEs regulate mammalian transcription, in terms of TE donation of enhancers and promoters, modification of 3D chromatin architecture, as well as generating novel regulatory genes, we refer readers to a recent review (Fueyo et al., 2022). It would be worth assessing how much of the regulatory network arose from TEs. Coupled with this, the functional impact of TEs on gene regulation still requires experimental verification.

## Systematic Functions of Transposable Elements

On top of the molecular functions of TEs, researchers are interested in how these molecular functions translate to the organismal level. In a recent study using natural populations of *Drosophila melanogaster* from five climatic regions, TEs were shown to be a likely contributor to an adaptation to the environment across evolution (Rech et al., 2022). This role in helping the species adapt to environmental conditions was similarly discovered for fish diadromy, which is the migration between freshwater and the sea, in a study that investigated genomes of 24 fish species (Carotti et al., 2021). Similar adaptions have also been observed for the migratory phenotype of birds such as willow warblers (Caballero-López et al., 2022), and in the relationship between sociality and genomic architecture in snapping shrimps, *Synalpheus* (Chak et al., 2021). Further, ancestral state construction suggested that TEs propagated the transition to eusociality (Chak et al., 2021). How and when TEs became domesticated and beneficial for the host has been discussed (Almeida et al., 2022), but this is beyond the scope of this review. The evolutionary routes by which TEs became domesticated, and which types of TEs are more prone to be domesticated are still unknown.

Across species, TEs are poorly conserved evolutionarily (Almeida et al., 2022). Scholars speculated the stochastic mobility of TEs across the genome contributed to this, and that many TEs may have derived from ancient spontaneous viral infections (Almeida et al., 2022). It remains largely enigmatic however, how TEs accumulated and diversified across species. Some postulated the role of population size in this process, which affects the efficiency of selection (Wells and Feschotte, 2020). However, this only explains part of the story because there is variation within species in the same taxonomic order (Wells and Feschotte, 2020). TE diversity therefore, could help explain the difficulties associated with translating animal studies to clinical applications (Bartlett and Hunter, 2018).

Coupled with this, variations in TEs are also present within a species, which may give rise to individuality (Mills et al., 2007; Bartlett and Hunter, 2018). This variation could emerge even in genetically identical animals due to the non-shared environments including random social encounters, individual history of experience, and self-made environments (e.g., nests) (Kempermann, 2019). The inter-individual variation in TEs is greater than traditional polymorphisms in protein-coding genes; and may explain different responses to stressors in different individuals (Lapp and Hunter, 2019).

## Transposable Elements and Epigenetics

The regulation of TEs is an area of high research interest (Figure 1A), particularly epigenetic regulatory mechanisms, which is a broad term that loosely defines processes such as transcription factor (TF) binding, DNA methylation, histone modifications to nucleosome positioning, alternative splicing and ncRNAs (Campbell and Wood, 2019).

Histone modifications alter the ability of molecules to bind and initiate transcription, and most modifications are methylation, acetylation and phosphorylation (Campbell and Wood, 2019). In one study using chromatin immuno-precipitation, histone 3 lysine 9 trimethylations predominantly localized to retrotransposons in the hippocampus (Hunter et al., 2012). Further, this was associated with repressed retrotransposon expression including intracisternal A particle (a type of ERV) and B2 (a type of SINE) (Hunter et al., 2012). Additionally, histone acetylation also regulates TEs. This was depicted in the enrichment of histone 4 lysine 16 acetylation, and histone 3 lysine 122 acetylations for LINE1 (Pal et al., 2022). While most epigenetic mechanisms are associated with silencing TEs, in this case, histone acetylation facilitated TE transcription (Pal et al., 2022). The mechanisms of these how histone modifications regulate TEs are beginning to be understood. For instance, histone 3 lysine 9 trimethylation depositions on TEs are mediated by activating TF 7 interacting protein in CD8 + T cells (Sin et al., 2022). Another type of trimethylation, histone 3 lysine 27 trimethylation, is deposited on TEs by Polycomb Repressive Complex 2, a mechanism that appears widespread across eukaryotes (Déléris et al., 2021).

Aside from histone modifications, TEs have a close relationship with ncRNAs (Fort et al., 2021). Most functional ncRNAs such as lncRNAs contain remnants of functionally inert TEs (Hunter, 2020). In the zebrafish genome, major fractions of miRNA binding sites are SINEs (Scarpato et al., 2015). Another class of ncRNAs important for TEs are piwi-interacting RNAs (piRNA). They act on TEs by triggering histone modifications *via* P-element Induced WImpy testis (PIWI) proteins or triggering transcript degradation through Argonaute proteins (Dechaud et al., 2019). A recent study unearthed further details of this interaction in *Paramecium* whereby PIWI proteins regulate TEs through Polycomb Repressive Complex 2 (Miró-Pina et al., 2022).

In tandem with ncRNAs, DNA modifications, by the same token, regulate TEs (Lapp and Hunter, 2019). One main type of DNA modification is DNA methylation, which occurs mainly on the cytosine bases of DNA (often on cytosine-guanine dinucleotide (CpG) through methyltransferases DNA methyltransferase (DNMT) 1, 3a and 3b, to decrease transcription, respectively (Deniz et al., 2019). Scholars theorized that 5-methylcytosine modification in the DNA has developed specifically to silence TEs in many species (Deniz et al., 2019). Other DNA modifications that regulate TEs include DNA modifications on N6-methyladenine and oxidative derivatives of 5-methylcytosine (Deniz et al., 2019).

The epigenetic relationship with TEs is a two-way street. Whilst TEs are regulated by epigenetics, epigenetic activities are also regulated by TEs. An example of this is how TEs regulate transcription by modifying the 3D chromatin architecture (Fueyo et al., 2022). One way to measure this is using Hi-C, which is a high throughput technology to capture chromatin conformation by measuring how two DNA fragments physically associate in 3D space (Kong and Zhang, 2019). From Hi-C results, L1 LINE and B1/Alu SINE RNAs appeared to be vital players in structuring the 3D genome (Kruse et al., 2019). Restructuring the chromatin, in terms of how accessible it is, can silence TEs, as in the case of microrchidia proteins may regulate chromatin accessibility of ERVs to impart TE silencing (Desai et al., 2021).

One last epigenetic mechanism we focus on vis-à-vis TEs are stress granules. In response to stress, stress granules are formed to assemble mRNAs and proteins (Chesnokova et al., 2022). The artificial inhibition of stress granule formation increased open reading frame 1 protein (ORF1p), an RNA-binding chaperone encoded by L1 (Chesnokova et al., 2022). This negative role of stress granules on ORF1p was, in the same fashion, observed in the context of hepatitis C virus infection (Schöbel et al., 2021). In tandem with these, this appeared to be the case in cancer cells too. Applying paclitaxel to breast cancer cell lines induced stress granules, which recruited and stabilized L1 (Shi et al., 2021). Stress granule formation is controlled by the cellular antiviral protein, myxovirus resistance protein B, which is correlated with restricted mobility of L1 (Huang et al., 2022). How these stress granules target TEs has been riveting to researchers. One study discovered this occurred through m^6^A RNA methylation of TEs (Fan et al., 2022).

## Role of Transposable Elements in Development

Several lines of evidence have demonstrated dynamic changes in TE transcription during embryonic development, and have hinted that TEs may drive important aspects of development (Kruse et al., 2019; Figure 1D). Specifically, during the pre-implantation stages of mouse embryogenesis, converging evidence from the past 20 years has concluded that TE activation during this stage is crucial (Hermant and Torres-Padilla, 2021). Embryogenesis requires the proliferation of embryonic stem cells (ESC) (Pontis et al., 2019). In humans, TEs regulate the transcription network of ESCs (Pontis et al., 2019). The transition of ESCs past the totipotent 2-cell stage is driven by TEs such as L1 (Percharde et al., 2018). In murine ESCs, the TE, Murine Endogenous Retroviral Element, facilitated the transition of ESC to the 2-cell stage by reorganizing the chromatin (Kruse et al., 2019). This mechanism likely occurs in human and across all stages of embryogenesis according to research showing more than one-third of open chromatin in the human embryo during pre-implantation were embedded in or contained TEs (Pontis et al., 2019). Additional mechanisms are being uncovered, such as the totipotency established by L1 and ERV, which are regulated by the TF, orthodenticle homeobox 2, through activating homeobox protein (Guo et al., 2021). Going past the 2-cell stage, TE expression was up-regulated during the 4-cell stage for SINE-VNTRAlu (SVA), Human ERV type K and type H (HERV and HERVH) subgroups (Pontis et al., 2019). These and other findings imply that TEs may be fundamental players in the development of multicellularity, in general (Hunter, 2020).

Trophoblasts are important cells in the blastocyst that feeds the embryo with nutrients (Todd et al., 2019). Using mouse ESCs and trophoblast stem cells, researchers found LTR with enhancer-like properties that drove gene expression specific to each cell type (Todd et al., 2019). This suggests TEs could impart tissue specificity. Bolstering this idea, during E14.5 and P0 in mice, tissue-specific expression of TEs was observed in the intestine, liver, lung, stomach, and kidney (Miao et al., 2020). This tissue-specificity of TE expression was commensurately ferreted during zebrafish embryogenesis using single-cell RNA-sequencing, including stage-specific expression (Chang et al., 2022). Tissue-specific expression of TEs suggests TEs may regulate the development of specific tissues. This was espoused for brain development where L1 acted as gene regulatory elements for genes responsible for synaptic transmission and cell communication such as cadherin-8, proto-oncogene receptor tyrosine kinase, Synaptotagmin 1, TBC1 Domain Containing Kinase, phospholipase C beta 1, in a neural progenitor cell model (Jönsson et al., 2019). One study suggested that TEs may regulate brain development through epigenetic mechanisms (Playfoot et al., 2022). The study examined brain development across childhood and adolescence in humans and saw distinct spatiotemporal dynamics of TE-embedded miRNA expression (Playfoot et al., 2022). Supplementary to miRNA, DNA methylation also regulates TEs during development (Haggerty et al., 2021). This has been found using methylation-depleted mouse ESCs, where DNMT1 catalyzed DNA methylation at retrotransposons (Haggerty et al., 2021). For a detailed overview of the role of TEs during events such as zygotic genome activation, cell pluripotency, gastrulation, and maternal-fetal interactions, we refer readers to a recent review (Senft and Macfarlan, 2021). Beyond the role of TEs on embryonic tissue, we are allured with their roles in extra-embryonic tissue including placental and maternal tissue, which would require first unearthing the cell types at this maternal-fetal interface.

There is a substantial amount of literature on the epigenetic processes that govern early development. For example, DNA methylation plays a role in the parent of origin expression seen in imprinting, where parent-specific allele patterns of DNA methylation can be found at imprinted sites in the germ cell (Tucci et al., 2019). In this context, DNA methylation acts as a marker enabling the identification of parent-specific gene copies and thus, which gene to express. Histone modifications also play important roles, for example, blocking histone 3 lysines 27 trimethylation is a critical part of the imprinting of paternal alleles and modulated fetal growth (Tucci et al., 2019). The genomic profile of developing organisms is also critically reliant on early developmental methylation patterns, and the early establishment of these methylation patterns is necessary for the constitution of chromatin structure (Hashimshony et al., 2003). Researchers could use this understanding of epigenetics in development to disentangle the role of TEs. Some unsettled questions include how TE silencing is maintained during development, and the compensatory mechanisms to prevent TE mobility when epigenetic silencing is compromised.

## When Transposable Elements Go Awry

Given that TEs activities are critical during development, it is not surprising that deleterious TE events are associated with developmental disorders. This is clearly corroborated in different diseases and disorders related to TE dysregulation in developmental and other contexts (Kazazian and Moran, 2017; DeRosa et al., 2022). For example, variations in L1 expression in cord blood and placental tissue are associated with infertility (Barberet et al., 2022). TEs could propel diseases and disorders by inserting into exons that disrupt functional genes. To mention a few, in hemophilia, L1 is inserted into favor VIII locus on chromosome X (Kazazian et al., 1988; Payer and Burns, 2019). In retinoblastoma, L1 is inserted into the Retinoblastoma Transcriptional Corepressor 1 gene, causing changes in mRNA splicing (Payer and Burns, 2019). Complementary to retinoblastoma, which is a type of neoplasia, the DNA damage that L1 expression foments, could contribute to other types of malignant transformation and oncogene-induced senescence (Burns, 2020). Additionally, TEs may facilitate inflammatory diseases. In the inflammatory disorder, Aicardi–Goutieres syndrome, Alu elicits an interferon response that drives the progression of this disorder (Burns, 2020).

Recently, genome-wide association studies (GWAS) deployed the partition heritability method to understand the genetic architecture of diseases including TEs (Hormozdiari et al., 2019). From GWAS data on 41 independent diseases and complex traits, including autism spectrum, depressive symptoms, neuroticism, schizophrenia, and age first birth, it was determined that TEs correlated well with the heritability of these diseases and traits (Hormozdiari et al., 2019). Many of these diseases were mental disorders, and specific studies have delved into how TE dysregulation imparts influence on neuropsychiatric diseases (Misiak et al., 2019; DeRosa et al., 2022). For example, in children with an autism spectrum disorder (a neurodevelopmental disorder), HERVH is overexpressed in the peripheral blood mononuclear cells (Misiak et al., 2019). Researchers have also shown a role of L1 in idiopathic autism in several brain regions including an elevated L1 level in the cerebellum (Misiak et al., 2019). In tandem with TEs, transfer RNAs also partake in the etiology of neurodevelopmental disorders (Blaze and Akbarian, 2022). TEs have been associated with shorter transfer RNA-derived RNA fragments in plants (Zahra et al., 2021), which are known to inhibit translation and modulate immune response (Blaze and Akbarian, 2022). Therefore, this may be another avenue worth exploring for TE pathogenesis in developmental diseases and disorders.

## Sexual Transmission of Transposable Elements

For TEs to persist in a species, they require vertical transmission through sexual reproduction. This results in the generation of offspring that continue to harbor these TEs. However, hosts also have an interest in regulating and repressing TE activity in the germline, and a number of mechanisms, such as piRNAs exist to do so (Wang and Lin, 2021). The importance of sexual transmission of TEs cam be observed in studies where asexual species exhibits less TE load. For example, in asexual arthropod lineages of crustaceans (e.g., insects and mites), there were less TE load compared to sexual lineages (Bast et al., 2016). By the same token, a recent study on asexual yeast, *Saccharomyces cerevisiae*, found a reduction of TE loads across asexual reproduction likely to be due to an increased level of TE excision (Bast et al., 2019).

During sexual reproduction, meiosis results in homologous recombination and has been shown to interact with TEs in several species (Underwood and Choi, 2019). As an illustration, in humans, TEs can modify Histone-Lysine *N*-Methyltransferase, which regulates where the meiotic recombination occurs (Underwood and Choi, 2019). Additionally, the LTR, Ty3/Gypsy co-opts the binding site of the meiotic TF, NDT80, in the budding yeast *Saccharomyces cerevisiae* (Laureau et al., 2021). This ensured the propagation of Ty3/Gypsy in the germline (Laureau et al., 2021). Expression of TEs in the germline was noted in embryonic male mice germ cells (Yang et al., 2020) in addition to the testis and ovaries of the black tiger shrimp *Penaeus monodon* (Sukthaworn et al., 2020). In the Japanese medaka, *Oryzias latipes*, TE expression in the germ line was developed by TE hijacking the regulatory sequence of neighboring sexual genes (Dechaud et al., 2021). In accordance with this, *Drosophila* ovary germline stem cells also express TEs (Story et al., 2019). This germline TE expression was higher than TE expression in somatic support cells (Story et al., 2019), conforming to the idea of vertical transmission as being the preferred transmission of TEs.

Given the importance of sexual reproduction in the persistence of TEs, scholars have speculated that complementary to hijacking germline machinery for their own propagation, TEs may also participate in regulating functions in the germline (Dechaud et al., 2019). Epigenetic mechanisms, namely piRNAs, DNA methylation, histone modifications, and repressor proteins have the ability to regulate TEs in the germline (Story et al., 2019; Miller et al., 2020; Sukthaworn et al., 2020; Yang et al., 2020). Apropos to the widespread expression of TEs across species, we are captivated by the question of the stringency of epigenetic silencing, and how this advanced the success of TEs in colonizing genomes.

## Sexual Development

In accordance with the evidence of TE activity in the germline, and their higher levels in sexually reproducing species, TEs may also be involved in sexual development. Prior to discussing the role of TEs in sexual development, we provide a brief introduction to the process of sexual development. Because of the diversity of sexual development across species, we focus primarily on humans and rodents. Sexual development differs across species ranging from environmental sex determination to genetic sex determination, and different sex chromosome systems including X, Y, Z and W (Dechaud et al., 2019).

For mammals, sex determination occurs where undifferentiated gonads are mainly made up of primordial germ cells and somatic progenitors (around E10.0 in mice) that can either develop into testes or ovaries (Stévant and Nef, 2019). One study that deployed time series single-cell RNA sequencing identified that the formation of these somatic precursors occurs through a similar transcriptomic program between the sexes, but the differences in temporal expression may give rise to the sex differences (Stévant et al., 2019). Following that, sex determination happens when somatic progenitors commit to either pre-Sertoli cells in males or pre-granulosa cells in females depending on their sex chromosomes around E11.5 (Stévant and Nef, 2019). From Assay for Transposase-Accessible Chromatin using sequencing and chromatin immunoprecipitation sequencing studies, they found that the chromatin landscape of the somatic precursor cells digress as they commit to either a male or female fate (Garcia-Moreno et al., 2019). In tandem, alternative splicing was unearthed to be an important regulatory mechanism in sex determination (Planells et al., 2019).

The point of divergence into a male or female “path” in a bipotential embryo begins with an expression of sex-determining region Y (SRY) which, when expressed, commences the male path by triggering the formation of testes. The testes then produce Anti-Mullerian hormone (AMH) and eventually testosterone, which further reinforces this male-typical developmental path. Females, on the other hand, lacking both SRY and AMH, also lack this increased gestational exposure to testosterone. Females also lack exposure to estradiol from the *in utero* environment due to elevated levels of alpha-fetoprotein which isolates the estradiol in circulation around the embryo (Morris et al., 2004; McCarthy and Arnold, 2011; Wu and Shah, 2011). Thus, the embryonic male appears to develop in the presence of circulating gonadal steroids while the female develops in the absence of these steroids. Embryo testosterone production remains contentious, so most of these circulating gonadal steroids are speculated to be of maternal origin (Holland et al., 2019). Therefore, in addition to embryonic exposure to testosterone, the effect of testosterone continues postnatally through neonatal production of testosterone (Clarkson and Herbison, 2016). This alludes to the now widely accepted hypothesis surrounding this sex difference in the neonatal hormonal milieu, where exposure to hormones during gestation has “organizational” effects, and later exposure (like during puberty) has “activational” effects (Phoenix et al., 1959).

As an illustration of how this progress in specific systems, we focus here on brain development. Both organizational and activational actions of hormones are utilized to describe how the brain and behavior are sculpted at different points in development *via* gonadal steroids. Neonatal surges of testosterone are well-documented to be pivotal and sufficient for sexual differentiation in the brain (Clarkson and Herbison, 2016). Evidence from rodent studies shows that the production of testosterone in the early postnatal period masculinizes selected brain regions (McCarthy, 2020). In the human male, this testosterone surge peaks around 3 months of age (Bakker, 2022). Sexual differentiation of the brain is critically dependent on timing. For example, the time by which a neonatal rodent can be permanently masculinized or feminized with exogenous gonadal hormone only extends approximately up to the first week of life (McCarthy and Nugent, 2015). In support of this, a recent work constructed a comprehensive map of genomic estrogen receptor-alpha (ESR1) binding sites in the brain (Gegenhuber et al., 2022). This revealed the role of ESR1 in masculinization through establishing specific neuron types and long term activation of gene expression (Gegenhuber et al., 2022).

Some of the brain regions known to have size differences between sexes are driven by gonadal steroids (McCARTHY, 2008; McCarthy et al., 2017). For example, regions in the central nervous system such as the spinal nucleus of the bulbocavernosus, the anteroventral periventricular nucleus of the hypothalamus, the medial preoptic area (POA), and the bed nucleus of stria terminalis (BNST) are larger in adult males than in females (McCarthy, 2020). Masculinizing these morphological differences in females can be achieved through neonatal exposure to testosterone, which acts on the apoptotic pathways that establish these differences (Zup et al., 2003; Forger, 2006; Vries, 2008; McCarthy, 2016; Wu and Tollkuhn, 2017).

## Transposable Elements on Sex Chromosomes

Several lines of evidence support the idea that TEs are involved in sexual development. Prior, we discussed the expression preference in the germline by TEs in support of their interest in vertical transmission. To corroborate that TEs participate in sexual development, TEs should be expressed in sex chromosomes. This was indeed present in many species. One example is the half-smooth tongue sole *Cynoglossus semibreves*, which possess well-differentiated sex chromosomes, with a large W chromosome (Wang et al., 2014). Using pyrosequencing of cDNA samples, researchers uncovered in the heterochromatic regions of the W chromosome, TEs were highly elevated (Wang et al., 2014). Accompanying this, from whole-genome sequencing and transcriptomic data in *Drosophila*, researchers revealed that two-thirds of genes in the male Y chromosome scaffold exhibited sequence similarity with TEs (Meisel et al., 2017). TE expression in sex chromosomes is likewise observed in humans (Tang et al., 2018). In fact, the Y chromosome has a higher density of TEs in comparison to autosomes, namely 30 times higher for LTR and four times higher for Alu and L1 (Tang et al., 2018). There are postulations that TEs may be involved in the differentiation of the sex chromosomes themselves (Srikulnath et al., 2022). Ty3/Gypsy TE insertion into W/Y chromosomes achieved non-homology/non-recombination (Srikulnath et al., 2022). This is bolstered by genome-wide comparative Single nucleotide polymorphisms studies from several fishes and reptiles that a number of sex-related loci exhibited similarity to Typ3/Gypsy (Srikulnath et al., 2022). There are no active Ty3/Gypsy retrotransposons known in mammals but several sequences are present in Ty3/Gypsy (Laureau et al., 2021).

We noticed a paucity of studies on the function these TEs perform in the sex chromosomes, but a few have been proposed. One is dosage compensation, which is an idea that corresponds to the degeneration of the Y chromosome (Muyle et al., 2021). This results in insufficient gene dosage across the male XY chromosome (Muyle et al., 2021). Dosage compensation is a process whereby the sole X chromosome of the male upregulates gene expression to compensate for the absence of the other X chromosome (Muyle et al., 2021). The TE, Helitron in the X chromosome of *Drosophila Miranda*, facilitates dosage compensation by incorporating several male-specific lethal complex binding sites (Ellison and Bachtrog, 2013). The recruitment of male-specific lethal complex to the X chromosome in males increases transcription (Muyle et al., 2021). It’s unclear whether Helitron regulates dosage compensation in other species, such as Helraiser (part of the Helitron family) present in the little brown bat, *Myotis lucifugus* (Kosek et al., 2021), or in human cells (Sandoval-Villegas et al., 2021). Supplementary to dosage compensation, TEs function as a chromatin remodeler in the mammalian X chromosome (Chatterjee, 2017). The X chromosome could confer diverse phenotypes due to heterochromatin spreading capacitated by TEs (Chatterjee, 2017). Such functions can be broadly described as epigenetic, but a substantial amount of work needs to be done to disentangle these functions. Hints from existing knowledge of epigenetics in sex chromosomes could help shed light on other functions of TEs on sex chromosomes.

X-chromosome inactivation is regulated by epigenetics (Knoedler and Shah, 2018). The inactivation of one of the X chromosomes in females needs to occur to account for the excess genetic material, and so this process ensures females only have one functional copy of the inherited genes, although some genes have the capacity to escape inactivation. This process first involves selecting which X to silence, and then subsequently maintaining this silencing (Giorgetti et al., 2016). Portions of the X inactivation center modulate chromosomal counting and transcription of the lncRNA, X-inactive specific transcript (*XIST)*. While not every mechanism has been extrapolated, X inactivation is driven by *XIST* chromosomal wrapping, enzymes involved in histone and DNA modifications, as well as compaction of the inactive X (Lu et al., 2017). Acetylation of histone 3 lysine 9 and methylation of histone 3 lysine 4 occur following *XIST* wrapping in embryonic mouse cells, but these events occurred before actual X inactivation (Okamoto et al., 2004), suggesting that these modifications are more directly involved in mediating X inactivation once *XIST* is in place. In tandem with that, histone deacetylases, the enzymes that make DNA less available to TFs, by the same token, participate in X inactivation (Zylicz et al., 2019). Specifically, researchers found the necessary and sufficient role of histone deacetylase 3 on X chromosome silencing upon *XIST* wrapping, where histone deacetylase 3 action leads to silenced genes (Zylicz et al., 2019).

## Sex Differences in Transposable Elements

In addition to TE expression in sex chromosomes, the sex differences in TEs further imply their potential roles in sexual development. In a rat RNA-sequencing study that examined several organs including the brain, gonads, and liver across 4 developmental stages (2, 6, 21, 104 weeks old), there were sex differences in LTR expression, especially in the kidney and the liver (Dong et al., 2017). Their development data suggest that some differentially expressed TEs arose during adolescence (Dong et al., 2017). In another species, the Japanese medaka, *Oryzias latipes*, TEs overexpressed in the testis compared to ovaries (Dechaud et al., 2021). From these, we realized a scarcity of studies that examined sex differences in TEs, and pose a major avenue for future research.

Aside from sex differences in TE expression, TEs could relate to sex differences through epigenetics. To mention a few, we highlight a study deploying CpG methylation quantification from whole-genome bisulfite sequencing data in the tilapia, *Oreochromis spp*. (Wan et al., 2016). Most of the TEs were highly methylated, and were distinct between males and females (Wan et al., 2016). In *Drosophila melanogaster*, TEs were proposed to drive differences in heterochromatin/euchromatin balance, considering that females harbored a higher density of TE in the heterochromatic region (Chatterjee, 2017). Seeing that piRNAs predominantly function to regulate TEs (Bartlett and Hunter, 2018), it is possible that sex differences in piRNA-related systems would be germane to TEs. In the silkworm, *Bombyx mori*, females displayed a biased expression of PIWI (Zhao et al., 2011). Furthermore, in the gonad of the olive flounder, *Paralichthys olivaceus*, PIWI-related gene expression was 2,000 fold higher in the testis compared to the ovary (Zhang et al., 2016). Similar differences in PIWI-related genes were corroborated in the gonads of turbot (*Scophthalmus Maximus)*, snakeskin gourami *(Trichopodus pectoralis*), and yellowfin seabream (*Acanthopagrus latus*) (Wang et al., 2018; Boonanuntanasarn et al., 2020; Li et al., 2020).

## Sex Differences in Epigenetics

Determining sex differences in epigenetic mechanisms could be helpful in uncovering the role TEs in the development and maintenance of sexually dimorphic organs such as the brain. Epigenetic events in the brain are both regulated by age and sex. For example, at P1, female rats exhibit increased DNMT3a mRNA in the amygdala compared to males, but this sex difference disappeared at P10 (Kolodkin and Auger, 2011). In addition, both estradiol and dihydrotestosterone treated P1 females showed decreases in DNMT3a mRNA like that seen in males. Together, these results provide a timeline by which epigenetic marks may change as males and females age, but it will be up to future studies to determine the potential functional role of these differences. Interestingly, development of the amygdala is typically expedited in both rodent and human females (Mizukami et al., 1983; Uematsu et al., 2012). Thus, the investigation into a potential relationship between sex differences in DNMT3a mRNA and the developmental trajectory of the male and female amygdala may be warranted. Unlike the amygdala, DNMT3a expression had no differences in gene expression and enzyme activity in the ventromedial hypothalamus, POA, or hippocampus throughout postnatal development and into adulthood in C57BL/6 mice (Cisternas et al., 2020). To our knowledge, no other studies examined DNMT3a in the amygdala, which was done in Sprague–Dawley rats using qPCR and Western blotting (Kolodkin and Auger, 2011). Henceforth, additional research is needed to leverage technologies with higher resolutions, such as spatial transcriptomics (Liu et al., 2021), and single-cell RNA-sequencing (Ng et al., 2021). One major outstanding question is whether or not these epigenetic marks are reversible and what factors drive them (Arnold, 2020).

Differences in epigenetic markers have also been reported in brain regions that are known for being hubs of morphological and functional sex differences. For example, in neonate male mice, Tet enzyme (methylcytosine dioxygenases that remove DNMTs) increased in the POA and ventromedial nucleus of the hypothalamus (Cisternas et al., 2020). This study also found testosterone treatment to slightly masculinize Tet expression in females. This demonstrates that this sex difference in Tet enzyme and hormones may work together to establish these differences. Sex differences in histone 3 lysine 4 trimethylation have also been found in the mouse BNST and POA (Wu and Tollkuhn, 2017). Furthermore, a major (71%) increase in histone 3 lysine 4 trimethylation peaks in adult female mouse BNST and POA compared to males (Shen et al., 2015). Genes associated with these increased peaks were found to be related to synaptic transmission (Shen et al., 2015). These results further demonstrate baseline sex differences in epigenetic marks while also suggesting that these differences are maintained over time since the researchers utilized adults. In support of this, exogenous estradiol treatment in ovariectomized mice has been shown to increase histone modifications and alter chromatin structure in POA and ventromedial hypothalamus (Gagnidze et al., 2013), further demonstrating that activational effects of estrogen later in adulthood may be happening through epigenetic mechanisms. Two GWAS also found sex differences in methylation in BNST and POA, where females seem to have overall higher levels of DNA methylation and more methylated CpG sites compared to males. In addition, these differences are partially driven by hormones, as neonatal treatment with estradiol attenuated DNMTs and thus, DNA methylation in developing POA at CpG sites (Ghahramani et al., 2014; Nugent et al., 2015).

A sex difference in the epigenetic landscape is supported by other studies, notably in female mice across the estrous cycle, neuronal chromatin organization in the ventral hippocampus fluctuates which corresponded with transcriptional changes mediated by Early Growth Response 1 (Jaric et al., 2019). Sex differences have similarly been reported in miRNAs which modulate gene expression through their ability to silence or interfere with mRNA (Wightman et al., 1993; Reinhart et al., 2000). Specifically, the majority of miRNAs assayed in one study from neonatal rats differed in expression based on sex (Morgan and Bale, 2011). After blocking the aromatization of testosterone into estradiol in neonatal males, miRNA levels became indistinguishable between males and females. This study ultimately demonstrates a link between estradiol and miRNA. Therefore, hormones could drive differences in miRNA, which may modulate differential gene expression. The intimate relationship of TEs with miRNA (Playfoot et al., 2022), insinuates that TEs engage in this process.

## Transposable Element Regulation of Sexual Development-Related Genes

In parallel to sex chromosomes and sex differences, TE regulation of sexual-development genes is another avenue of evidence that buttress the role of TEs in sexual development. One of these genes includes Doublesex and Mab-3 Related TF 1, which participates in sex determination and differentiation among a wide range of species from corals, flies, fish and mammals (Herpin et al., 2010). For medaka fish (*Oryzias latipes*), Doublesex And Mab-3 Related TF 1, acts as the master regulator of male-sex determination, and its regulatory element upstream of the gene is within a TE (Herpin et al., 2010). Another gene is SRY, which is pivotal for male sex determination in mammals (Prokop et al., 2013). SRY in rats was found to co-express with L1 (Prokop et al., 2013). Up until a few years ago, SRY was considered a single-exon. Researchers then discovered a cryptic second exon in mice SRY, which through gene-deficient and ectopic expression studies, was deemed imperative for sex determination (Miyawaki et al., 2020). Their further analysis uncovered this exon evolved from TE activity that replaced the degron sequence, which would have otherwise degraded SRY as it was translated (Miyawaki et al., 2020). Next to SRY, another important factor in rodent and human sexual development is insulin and insulin-like growth factors (Neirijnck and Nef, 2019). In exon 5 of insulin-like growth factor 1 of nine mammalian species, researchers found a TE, mammalian interspersed repetitive-b element, in its genomic region (Annibalini et al., 2016).

As discussed previously, ESR1 is imperative for sexual development in part through their interactions with gonadal steroid hormones. Point mutations in ESR1 led to sexual developmental defects in the reproductive organs and mammary glands (Simond et al., 2020). One thing to note in relation to TEs is the pseudoexon of ESR1. Pseudoexons are sequences similar to an exon, but it does not take part in splicing (Vorechovsky, 2010). The pseudoexon of ESR1 possesses several components supplied by TEs: branchpoint sequence (involved in splicing), upstream region, and polypyrimidine tract (region promoting assembly of spliceosome) (Vorechovsky, 2010). These pseudoexons can modulate enhancers, silencers and other sequences that can alter the function of the gene (Vorechovsky, 2010). In relation to ESR1, estrogen response elements are binding sites for ESR1 to regulate transcription (Mason et al., 2010). One-third of these response element sequences have been found to reside most commonly with Alu (Mason et al., 2010). Luciferase reporter assays confirmed that these Alu sequences contribute to enhancer function which recruits ESR1 (Mason et al., 2010). Accompanying this, a recent RNA-sequencing study of human breast tumor samples (estrogen receptor-positive, human epidermal growth factor receptor 2 negative), when compared to matched normal tissue, several TEs were differentially expressed, and this correlated with ESR1 expression (Yandım and Karakülah, 2019). Another steroid receptor is the progesterone receptor, is also important for sexual development (Jacobsen et al., 2009). Just like ESR1, progesterone response elements regulate progesterone receptor-mediated transcription (Jacobsen et al., 2009). This response element contains a sequence that resembles Alu (Jacobsen et al., 2009). Whether editing these TE-related sequences in these response elements can affect sexual development remains to be explored. In tandem with gonadal steroid hormones acting through cognate steroid receptors like ESR1 for estrogen, hormones can also exert influence on sexual development through their presence in chromatin-modifying complex, and through interactions with signal transduction pathways (Knoedler and Shah, 2018). Altogether, this evidences underscores how TEs may be an integral part of gonadal steroid hormone mechanisms in sexual development.

## Future Studies on Transposable Elements, Sexual Development, and Epigenetics

To disentangle the causal link between TEs, sexual development and epigenetics, manipulation experiments are necessary. Insights can be attained from existing manipulation studies that test the causality between epigenetics and sex differences. For instance, using valproic acid to disturb histone acetylation, reduced the number of cells in BNST in males and in testosterone-treated females only (Murray et al., 2009). This sheds light on one possible mechanism of how testosterone masculinizes BNST (Murray et al., 2009). In addition, the inhibition of DNMT in neonatal female rates induced male-typical sexual behavior upon reaching maturity, suggesting that DNA methylation may not only initiate sex differences but also maintain them across development (Nugent et al., 2015).

These studies fall short in ascribing strong causal links because they use non-specific pharmacological agents to determine whether chromatin modifications are involved in sexual differentiation. It is highly plausible that these agents act indirectly through dysregulating the epigenome of an entire organism. Many advancements have been made that can help disentangle, more accurately and precisely, the role of TEs and their epigenetic regulation in in sexual development. To name a few, prime editing and piRNA-mediated interference can modulate or silence specific epigenetic-related genes (including TEs), respectively (Wannier et al., 2021; Priyadarshini et al., 2022). In addition, the role of specific cell types could be disentangled by cell ablation through bacterial nitroreductase systems (Sharrock et al., 2022). Subsequently, the effects of these manipulations can be investigated with high throughput techniques such as BOLARAMIS which allows spatial resolution of the transcriptome (Liu et al., 2021). Finally, the experiment can be carried out in model systems with high biological or clinical fidelity. Notably, TFs could convert easily accessible cells from the skin, urine, or hair of humans or wildlife, into any specific cell types of interest (e.g., neurons, oligodendrocytes etc.) (Ng et al., 2021).

Many interesting questions remain to be explored to closely examine the role of TEs in sexual development, but this requires a more fundamental understanding of TEs. What is the extent to which the epigenetic silencing of TEs is inherited? Could this inheritance differ between the sexes? Does TE domestication occur more frequently in some genomes due to their size? How does this relate to the sexual diversity seen across the tree of life? What are the polymorphisms associated with regulatory elements derived from TEs? Do these polymorphisms associate with within-species sexual diversity? What are the mechanisms do TE regulatory factors detect TE? Could disrupting these mechanisms result in altered sexual development?

## Concluding Remarks

The burgeoning of research on the deep genome has increased our understanding of TEs and their wide range of benefits. This benefit extends to the development of organisms, especially in the context of sexual development. We demonstrate the role of TEs in sexual development through the lens of sexual transmission, their presence in sex chromosomes, sexual differences in TE expression, and TE regulation of sexual development-related genes. Moreover, the epigenetic regulation of sex-specific development and gonadal steroid interactions with epigenetic processes support the role of TEs in this process, given the intimate interplay of epigenetic mechanisms and mobile genetic elements across eukaryotic evolution. While the area of TE in sexual development remains a largely unexplored topic, there are a myriad of future research opportunities are warranted. For example, sexual dimorphisms in TE expression should be studied in a variety of organs at different developmental stages in a variety of species including primates and humans. Furthermore, elucidating a comprehensive view of the sexual development gene network, and their interactions with TEs need further attention. All of the above also argues for a reassessment of the role TEs play not only in organismal development but in evolution and adaptation as well. It is now clear they are not simply junk but regulatory elements vital to their host’s ability to develop and survive.

## Author Contributions

VC and HD wrote the manuscript draft. All authors contributed to editing and revising the final version.

## Conflict of Interest

The authors declare that the research was conducted in the absence of any commercial or financial relationships that could be construed as a potential conflict of interest.

## Publisher’s Note

All claims expressed in this article are solely those of the authors and do not necessarily represent those of their affiliated organizations, or those of the publisher, the editors and the reviewers. Any product that may be evaluated in this article, or claim that may be made by its manufacturer, is not guaranteed or endorsed by the publisher.

## Abbreviations

AMH: anti-mullerian hormone
BNST: bed nucleus of stria terminalis
CpG: cytosine-guanine dinucleotide
DNMT: DNA methyltransferase
ESC: embryonic stem cells
ERVs: endogenous retroviruses
ESR1: estrogen receptor-alpha
GWAS: genome-wide association studies
HERV and HERVH: human ERV type K and type H
LINEs: long interspersed nuclear elements
lncRNAs: long non-coding RNAs
LTRs: long terminal repeat elements
POA: medial preoptic area
ncRNAs: non-coding RNAs
ORF1p: open reading frame 1 protein
PIWI: P-element induced wimpy testis
piRNA: piwi-interacting RNAs
SRY: sex-determining region Y
SINEs: short interspersed nuclear elements
SVA: SINE-VNTRAlu
TF: transcription factor
TEs: transposable elements
XIST: X-inactive specific transcript.
